# Midregional Proatrial Natriuretic Peptide (MRproANP) is associated with vertebral fractures and low bone density in patients with chronic obstructive pulmonary disease (COPD)

**DOI:** 10.1186/s12931-024-02902-2

**Published:** 2024-07-13

**Authors:** Franziska C. Trudzinski, Rudolf A. Jörres, Peter Alter, Henrik Watz, Claus F. Vogelmeier, Hans-Ulrich Kauczor, Subasini Thangamani, Manuel Debic, Tobias Welte, Jürgen Behr, Kathrin Kahnert, Robert Bals, Christian Herr, Claus Peter Heußel, Jürgen Biederer, Oyunbileg von Stackelberg, Sebastian Fähndrich, Emiel F. M. Wouters, Benjamin Waschki, Klaus F. Rabe, Felix J. F. Herth, Viktoria Palm, COSYCONET study group, Stefan Andreas, Kathrin Kanerth, Thomas Bahmer, Burkhard Bewig, Ralf Ewert, Beate Stubbe, Joachim H. Ficker, Christian Grohé, Matthias Held, Markus Henke, Anne-Marie Kirsten, Rembert Koczulla, Juliane Kronsbein, Cornelia Kropf-Sanchen, Christian Herzmann, Michael Pfeifer, Winfried J. Randerath, Werner Seeger, Michael Studnicka, Christian Taube, Hartmut Timmermann, Bernd Schmeck, Hubert Wirtz

**Affiliations:** 1 Department of Pneumology and Critical Care Medicine), German Center for Lung Research (DZL), Translational Lung Research Center Heidelberg (TLRC-H), Thoraxklinik University of Heidelberg, Röntgenstrasse 1, 69126 Heidelberg, Germany; 2 Institute and Outpatient Clinic for Occupational, Social and Environmental Medicine, German Center for Lung Research (DZL), LMU University Hospital, Ludwig-Maximilians-University (LMU), Comprehensive Pneumology Center Munich (CPC-M), Munich, Germany; 3 Department of Medicine, Pulmonary and Critical Care Medicine, German Center for Lung Research (DZL), Philipps University of Marburg (UMR), Marburg, Germany; 4 Pulmonary Research Institute at LungenClinic Grosshansdorf, Grosshansdorf, Germany; 5 Airway Research Center North (ARCN), German Center for Lung Research (DZL), Woehrendamm 80, 22927 Grosshansdorf, Germany; 6 Department of Diagnostic & Interventional Radiology, University Hospital of Heidelberg, Heidelberg, Germany; 7 German Center for Lung Research (DZL), Translational Lung Research Center Heidelberg (TLRC-H), Heidelberg, Germany; 8 Department of Pneumology, Hannover Medical School, Carl-Neuberg-Str. 1, 30625 Hannover, Germany; 9 Department of Internal Medicine V, CPC Comprehensive Pneumology Center, Member of the German Center for Lung Research (DZL), University Hospital, LMU Munich, Munich, Germany; 10 MediCenterGermering, Germering, Germany; 11 Department of Internal Medicine V - Pulmonology, Allergology, Critical Care Care Medicine, Saarland University Hospital, Homburg, Germany; 12 Department of Diagnostic and Interventional Radiology With Nuclear Medicine, Thoraxklinik, University Medical Center Heidelberg, Heidelberg, Germany; 13 Faculty of Medicine, University of Latvia, Riga, Latvia; 14 Faculty of Medicine, Christian-Albrechts-Universität Zu Kiel, Kiel, Germany; 15 Department of Pneumology, University Medical Centre Freiburg, Freiburg, Germany; 16 Department of Respiratory Medicine, Maastricht University Medical Center, Maastricht, The Netherlands; 17 Medical Faculty, Sigmund Freud University, Vienna, Austria; 18 Department of Internal Medicine, Sigmund Freud Private University, Vienna, Austria; 19 LungenClinic Grosshansdorf, Airway Research Center North, Member of the German Center for Lung Research, Pulmonary Research Institute, Woehrendamm 80, 22927 Grosshansdorf, Germany; 20 Department of Pneumology, Itzehoe Hospital, Itzehoe, Germany

## Abstract

**Background:**

Patients with COPD are often affected by loss of bone mineral density (BMD) and osteoporotic fractures. Natriuretic peptides (NP) are known as cardiac markers, but have also been linked to fragility-associated fractures in the elderly. As their functions include regulation of fluid and mineral balance, they also might affect bone metabolism, particularly in systemic disorders such as COPD.

**Research question:**

We investigated the association between NP serum levels, vertebral fractures and BMD assessed by chest computed tomography (CT) in patients with COPD.

**Methods:**

Participants of the COSYCONET cohort with CT scans were included. Mean vertebral bone density on CT (BMD-CT) as a risk factor for osteoporosis was assessed at the level of TH12 (AI-Rad Companion), and vertebral compression fractures were visually quantified by two readers. Their relationship with N-terminal pro-B-type natriuretic peptide (NT-proBNP), Mid-regional pro-atrial natriuretic peptide (MRproANP) and Midregional pro-adrenomedullin (MRproADM) was determined using group comparisons and multivariable analyses.

**Results:**

Among 418 participants (58% male, median age 64 years, FEV_1_ 59.6% predicted), vertebral fractures in TH12 were found in 76 patients (18.1%). Compared to patients without fractures, these had elevated serum levels (*p* ≤ 0.005) of MRproANP and MRproADM. Using optimal cut-off values in multiple logistic regression analyses, MRproANP levels ≥ 65 nmol/l (OR 2.34; *p* = 0.011) and age (*p* = 0.009) were the only significant predictors of fractures after adjustment for sex, BMI, smoking status, FEV_1_% predicted, SGRQ Activity score, daily physical activity, oral corticosteroids, the diagnosis of cardiac disease, and renal impairment. Correspondingly, MRproANP (*p* < 0.001), age (*p* = 0.055), SGRQ Activity score (*p* = 0.061) and active smoking (*p* = 0.025) were associated with TH12 vertebral density.

**Interpretation:**

MRproANP was a marker for osteoporotic vertebral fractures in our COPD patients from the COSYCONET cohort. Its association with reduced vertebral BMD on CT and its known modulating effects on fluid and ion balance are suggestive of direct effects on bone mineralization.

**Trial registration:**

ClinicalTrials.gov NCT01245933, Date of registration: 18 November 2010.

## Introduction

Among the reported multimorbidity found in patients with chronic obstructive pulmonary disease (COPD), osteoporosis is quite common [1–4]. This is partially related to the advanced age of many COPD patients [1], tobacco smoking [2, 5], and the treatment of acute exacerbations with systemic corticosteroids [6] which are known to promote the development of osteoporosis [7]. Inhaled corticosteroids might also play a role [8], and systemic inflammation could act as an additional risk factor. Although dual-energy X-ray absorptiometry (DXA) is the gold standard for determining bone mineral density (BMD) [5], there are also techniques for quantitative estimation based on the X-ray attenuation on routine CT scans (BMD-CT in Hounsfield-Units/HU) [9–11]. The presence of fractures can be detected based on semiquantitative or quantitative criteria [12], even if they are asymptomatic. Considering the prevalence of osteoporosis and the expenses required for its reliable diagnosis, it would be helpful to assess CT scans available from clinical routine for markers of osteoporosis, which afterwards could be substantiated by specific diagnostic procedures. Especially biomarkers could provide information about yet unrevealed, even subclinical, comorbidities, giving the opportunity for diagnosis and treatment in early stages.

We recently published data on the potential clinical value of cardiovascular blood markers as indicators of mortality risk in patients with mild to moderate COPD who had been diagnosed no more than 5 years ago [13]. In particular, the level of midregional pro-atrial natriuretic peptide (MRproANP) was linked to mortality in these COPD patients. Interestingly, two epidemiological studies reported an association between the cardiovascular marker MRproANP, as well as midregional pro-adrenomedullin (MRproADM), and the prevalence of fractures in the elderly [14], but without further assessment of bone characteristics or reference to COPD. The authors proposed neurological and hemodynamic factors as underlying mechanisms. In view of the adverse potential of systemic inflammation in COPD, mechanisms directly affecting bone density and integrity could also play a role, as some peptides modulating fluid and ion balance, such as MRproANP, have pleiotropic effects affecting not only the heart and hemodynamic system but also the kidney and endocrine system [15]. In addition, there is evidence that individuals with osteoporosis also experience an increased risk of coronary artery disease and stroke, even after controlling for confounding factors [16]. Conversely, congestive heart failure was found to be associated with increased bone loss and this was linked to elevated levels of human atrial natriuretic peptide [17]. Thus, there are several hints on a relationship between MRproANP and bone integrity.

We therefore wondered, whether a reduced vertebral density and the occurrence of vertebral fractures in patients with COPD, as early signs of osteoporosis, might be associated with cardiovascular markers that are often elevated in these patients. For this purpose, we used data from the COSYCONET (COPD and Systemic Consequences—Comorbidities Network) COPD cohort [18, 19], analyzing the role of several of these markers [13] in a subgroup of patients undergoing prospective chest CT scans that could be evaluated for vertebral density and fractures.

## Methods

### Study population

**Fig. 1:**
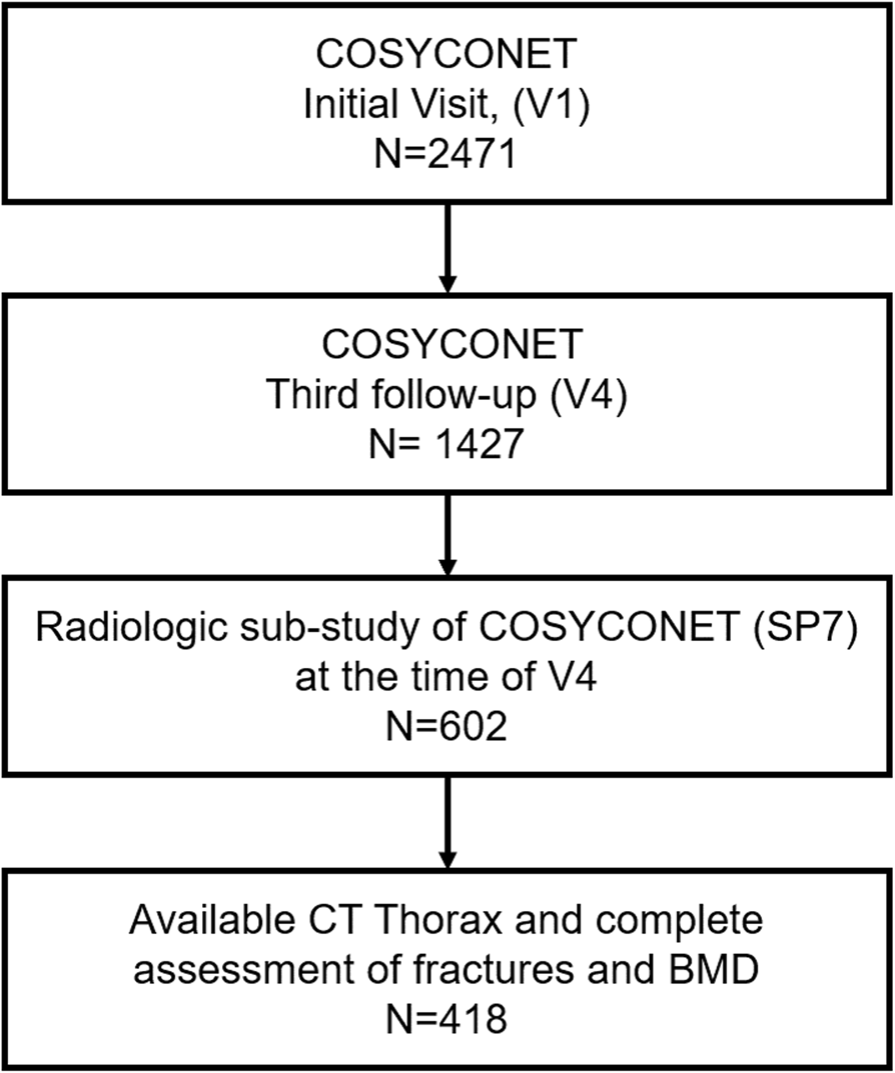
Flow chart of the patients included in the analysis. Of the 2,741 COSYCONET participants included at visit V1, *n* = 1,427 completed the third follow-up visit (V4). A subgroup of 602 patients underwent prospective CT scans at separate study visits at the time of V4. For the present study, the imaging data of 418 patients were analyzed for bone density and the presence of vertebral fractures (see Fig. 1). The functional and clinical data and biomarker measurements of the patients included in the current study were collected at visit 1 (V1)

Data from the prospective COPD cohort COSYCONET [19] was analyzed. This cohort enrolled 2741 patients, including patients diagnosed with Global Initiative for Chronic Obstructive Lung Disease (GOLD) grades 1–4, but also patients diagnosed with COPD that did not fit into GOLD 1–4, comprising the former grade 0 (COPD at risk) [20]. Inclusion and exclusion criteria, study protocol and assessments have been published earlier [19]; it should be noted that patients with a previous diagnosis of cancer, including lung cancer, were excluded. At about the time of the follow-up visit 4, i.e. 3 years after inclusion, a subset of patients underwent chest CT scans in inspiration and expiration in a substudy [19]. These scans were used in the present analysis to determine vertebral density of TH12 in Hounsfield-Units, as well as fractures. For inclusion we required complete imaging data of appropriate quality for these parameters; a corresponding flow chart is shown in Fig. 1. The COSYCONET study was conducted in accordance with the amended Declaration of Helsinki and approved by the Ethics Committees of the coordinating center Marburg and all study centers. Moreover, all participants gave their written informed consent. The study identifier at ClinicalTrials.gov is NCT01245933.

### Assessment of clinical and functional data

Age, body mass index (BMI), smoking status and pack years were determined as usual [19]. Spirometry and single-breath diffusing capacity for carbon monoxide were performed following the COSYCONET study protocol [19] and established recommendations [21–23]. For patients’ description and analysis, we used the forced expiratory volume in 1 s (FEV_1_), forced vital capacity (FVC) and the diffusing capacity of the lung for carbon monoxide (DLCO). The respective reference values were taken from the Global Lung Function Initiative (GLI) [24, 25]. In addition, 6-min walk distance (6-MWD) was determined and expressed in relation to reference values [26].

The categorization into COPD grades 1–4 was based on that proposed by GOLD for patients with a ratio of FEV_1_/FVC < 0.7 [27]. Patients with a ratio ≥ 0.7 were categorized as “at risk” (symbolized as “GOLD 0” in the results). The categorization into GOLD groups A/B/E [27] relied on the exacerbation history and the modified Medical Research Council (mMRC) scale [28]. The presence of comorbidities was derived from patient-reported, physician-based diagnoses [19], while medication was assessed via the approach that patients were asked to bring all their medication to the study visits [29]. In addition, the COPD assessment Test (CAT) [30], the St Georges’s Respiratory Questionnaire [31] and the International Physical Activity Questionnaire (IPAQ) [32] were used.

### Assessment of biomarkers

In all study sites, routine laboratory parameters comprised the blood levels of C-reactive protein (CRP), as well as that of creatinine, from which the estimated glomerular filtration rate (eGFR) was computed [33] in order to quantify kidney function. The concentrations of midregional pro-adrenomedullin (MRproADM) and midregional pro-atrial natriuretic peptide (MRproANP) in P100-stabilized plasma were determined using a Kryptor Compact Plus (BRAHMS GmbH, Hennigsdorf, Germany) in the central biobank (Homburg/Saarland), as well as the levels of B-type pro-natriuretic peptide (NT-proBNP; MILLIPLEX, Merck Millipore, Darmstadt, Germany). These markers were chosen for analysis as it could be argued that they were related to bone density and fractures on CT scans, due to their potential influence on bone metabolism [14, 34, 35]. We additionally determined a variety of other blood markers, particularly of inflammation, in order to use them as potential confounders. These included copeptin (COPAVP; BRAHMS GmbH, Hennigsdorf, Germany), osteopontin, Interleukin 6 (IL-6), Interleukin 8 (IL-8), tumor necrosis factor alpha (TNF), soluble receptor for advanced glycation end products (RAGE) (all Luminex Discovery Assay, R&D Systems, Abingdon, UK), and high-sensitivity troponin (HS-troponin; Architect STAT, Abbott Diagnostics, Wiesbaden, Germany).

### Assessment and analysis of CT scans

CT scans were performed following a standardized protocol [36] and submitted to an image data bank. Pseudonymized scans were analyzed with AIRC (AI-Rad Companion Chest CT VA30, Siemens Healthineers, Erlangen, Germany) on a dedicated work station.

**Fig. 2:**
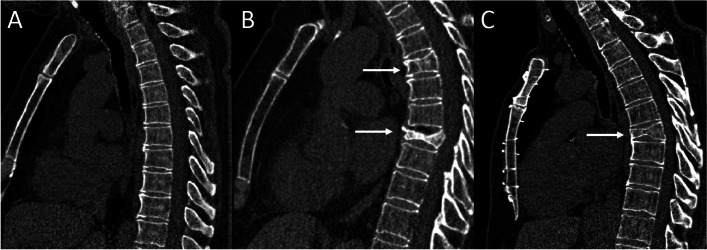
Examples of osteoporotic vertebral fractures in chest CT. **A** Sagittal view of the thoracic spine with minor degenerations, no vertebral body fractures (**B**) Osteoporotic vertebral body compression fractures in TH5 (Genant 1) and TH8 (Genant 3) (arrows). **C** Sintered and beginning wedge-shaped vertebra TH7 (arrow) with compression fracture Genant 2

Detection and location of vertebral compression fractures were visually assessed by a specialized, board-certified radiologist (> 5 years of experience) and a specially trained PhD-Student (> 1-year experience in CT spine analyses). Each fracture was visually graded via Genant [12] with respect to the height reduction into Grades 1 (20–25%), 2 (25–40%), 3 (> 40%) [12, 35]. Moreover, automated measurements of CT-derived bone mineral density (BMD-CT) performed by AIRC were expressed as mean attenuation in Hounsfield-Units (HU) for all thoracic vertebra (Fig. 2). For each participant, a sagittal image with analysis results was saved including the measurements. Automated data extraction from these images was performed with a Python script, with export of the measurement results in a CSV-file format. For analysis, the 12th thoracic segment of the spine (TH12) was chosen due to the fact that this could be evaluated best on the chest CT scans.

### Statistical analysis

Data in the tables are presented as percentages, or medians and quartiles. Group comparisons were performed with the Mann–Whitney U-test for metric variables, and Fisher’s exact test or chi-square statistics for categorical variables. The optimal cut-off values for the prediction of vertebral fractures with MRproANP and MRproADM were determined on the basis of receiver operating characteristic (ROC) curves and Youden indices, and the area under the curve (AUC) was computed to quantify the degree of association. The same analysis was performed for age as a risk factor.

A binary logistic regression analysis was performed to determine the association between the presence of vertebral fractures in TH12 and a set of predictors that were considered potentially relevant from a clinical perspective, as well as the biomarkers that had been found to show *p*-values ≤ 0.10 in univariate comparisons. The previous diagnosis of osteoporosis was omitted due to their triviality. The same set of predictors was used to identify variables associated with the TH12 BMD-CT mean attenuation in a linear regression analysis. Due to the availability of the cardiovascular markers, the values of all predictors were taken from the baseline visit V1, while the CT scans were performed 3 years after V1 at about the time of study visit 4. All statistical analyses were performed with SPSS (Version 29, IBM Corporation, Armonk, NY, USA), and a *p*-value < 0.05 was considered as statistically significant.

## Results

### Baseline characteristics

Overall, 418 patients (57.7% male) were included (Fig. 1), among whom 76 patients (18.2%) showed vertebral fractures and 342 (81.8%) no fractures. When analyzing the CT images, the two independent readers had a disagreement in the diagnosis of a compression fracture in only one case, resulting in an agreement of 99.76% from 418 Chest CTs (1/418) and 99.98% from 5016 examined vertebrae (1/5256).

As shown in Table 1, patients with vertebral fractures were significantly older than those without fractures and more frequently diagnosed with coronary artery disease and a diagnosis of osteoporosis (*p* < 0.05 each), while the use of oral corticosteroids was not significantly associated with fractures. With regard to other comorbidities, lung function, sex, BMI, smoking, clinical characteristics and questionnaire data, there were also no significant differences between the two groups, however, the TH12 mean attenuation differed (*p* < 0.001). Among the cardiovascular markers included, increased serum levels of MRproADM and MRproANP (*p* ≤ 0.005 each), but not of NT-proBNP, were found in patients with vertebral fractures. There were also no significant differences in the other biomarkers that had been analyzed as potential confounders (Table 2).

**Table 1:** Baseline characteristics of patients categorized by vertebral fractures detected on imaging

Variable	All *N* = 418	No vertebral fractures *N* = 342 (81.8%)	Vertebral fractures *N* = 76 (18.2%)	*P* value
Age (years)	64.0 (58.0, 69.0)	64.0 (57.0, 68.0)	68.0 (61.0, 72.3)	** < 0.001**
Sex (male/female	57.7%/42.3%	57.0%/43.0%	60.5%/39.5%	0.609
BMI (kg/m^2^)	26.5 (22.8, 30.1)	26.4 (23.2, 30.1)	27.0 (22.4, 30.5)	0.525
Pack years	44 (22, 74)	43 (23, 70)	51 (15, 93)	0.382
Smoking (active)	24.4%	25.4%	19.7%	0.376
Previous fracture (n/y)	4.5%	4.1%	6.6%	0.361
Hip fracture in parents	7.1%	7.2%	6.3%	1.000
Oral corticosteroids	8.6%	7.3%	14.5%	0.067
FEV_1_ (% predicted)	59.6 (46.9, 77.6)	59.5 (46.9, 77.3)	63.6 (46.9, 78.4)	0.956
FVC (% predicted)	84.3 (72.3, 96.1)	84.3 (73.1, 96.3)	84.5 (68.9, 94.9)	0.305
DLCO (% predicted)	61.9 (47.0, 76.7)	63.8 (46.6, 78.1)	60.8 (48.4, 74.8)	0.776
GOLD grade 0/1/2/3/4 (%)	15.3/11.8/40.8/25.2/7.0%	15.8/11.4/40.2/25.5/7.0%	13.2/11.4/40.2/25.5/7.0%	0.950
GOLD group A/B/E (%)	49.5/22.0/28.5%	49.1/22.5/28.4%	51.3/19.7/28.9%	0.867
SGRQ Total score	37.8 (26.5, 50.6)	37.3 (25.6, 50.0)	40.3 (28.2, 53.7)	0.153
SGRQ Activity score	52.2 (37.1, 67.6)	52.2 (36.7, 67.6)	56.9 (44.7, 71.9)	0.139
SGRQ Impact score	23.3 (11.3, 35.7)	22.7 (10.1, 34.9)	28.0 (13.9, 41,4)	0.153
SGRQ Symptom score	53.0 (37.2, 69.8)	53.0 (37.2, 70.0)	53.5 (37.1, 68.9)	0.615
CAT total score	17.0 (12.0, 21.0)	17.0 (12.0, 21.0)	17.0 (12.0, 23.0)	0.401
IPAQ score	3217 (1386, 6468)	3276 (1386, 6501)	2910 (1386, 6445)	0.685
6-MWD (% predicted)	71.7 (62.7, 79.9)	71.3 (62.7, 79.5)	73.4 (62.3, 81.7)	0.529
Diabetes	10.5%	11.4%	6.6%	0.301
Hypertension	49.8%	49.4%	51.3	0.801
Coronary artery disease (y/n)	13.6%	11.7%	22.4%	**0.025**
Heart failure (y/n)	5.7%	9.2%	5.0%	0.171
eGFR (mL/min)	86.8 (74.3, 94.8)	87.2 (73.9, 95.7)	85.1 (74.5, 90.9)	0.106
Osteoporosis	12.0%	8.8%	26.3%	** < 0.001**
TH12 mean attenuation (HU)	125.0 (94.0, 155.0)	128.0 (100.8, 158.0)	111.0 (83.5, 141.3)	** < 0.001**

Continuous variables are presented as median values and quartiles (in parentheses). Categorical variables are shown as percentages. Group comparisons were made using the Mann–Whitney U-test or Fisher’s exact test or the chi-square statistics, as appropriate. *BMI* body mass index, *FEV*_*1*_ forced expiratory volume in 1 s, *FVC* forced expiratory volume, *DLCO* diffusing capacity of the lung for carbon monoxide, *SGRQ* St George’s Respiratory Questionnaire, *CAT* COPD Assessment Test, *IPAQ* International Physical Activity questionnaire, *eGFR* estimated glomerular filtration rate. Please note that all variables refer to Visit V1, while the outcome of fractures and TH12 bone density refer to Visit V4 performed about 3 years after Visit V1

Numbers in bold represent *p* ≤ 0.05

**Table 2:** Levels of blood biomarkers categorized according to the presence of vertebral fractures detected on CT scans. Median values and quartiles (in parentheses) are given

Biomarkers	All *N* = 418	No vertebral fractures *N* = 342	Vertebral fractures *N* = 76	*P* value
MRproANP (pmol/l)	61.4 (44.8, 88.8)	58.6 (42.5, 83.8)	77.4 (59.9, 112.0)	** < 0.001**
MRproANP ≥ 65 nmol/l (%)	46.7%	41.5%	70.0%	** < 0.001**
MRproADM (nmol/l)	0.65 (0.56, 0.75)	0.64 (0.55, 0.75)	0.70 (0.62, 0.79)	**0.005**
MRproADM ≥ 0.60 nmol/l (%)	64.8%	61.7%	79.1%	**0.007**
NT-proBNP (pg/mL)	180.9 (32.6, 373.1)	171.8 (30.3, 386.2)	233.6 (39.0, 360.7)	0.449
COPAVP (pmol/l)	5.22 (3.54, 8.23)	5.15 (3.43, 8.21)	5.38 (3.64, 8.53)	0.315
Osteopontin (pg/ml)	3723 (2131, 6871)	3681 (2135, 6719)	3803 (1943, 9127)	0.318
HS-Troponin (pg/ml)	3.5 (2.3, 5.3)	3.4 (2.3, 5.0)	3.9 (2.6, 7.3)	0.207
RAGE (pg/ml)	839 (685, 1077)	856 (682, 1077)	791 (703, 1079)	0.230
TNF (pg/ml)	8.13 (4.83, 12.51)	8.17 (4.80, 12.32)	8.00 (4.87, 14.08)	0.391
IL-6 (pg/ml)	2.32 (0.30, 7.33)	2.29 (0.27, 7.37)	2.59 (0.63, 7.50)	0.913
IL-8 (pg/ml)	7.96 (5.17, 10.83)	7.92 (4.93, 10.66)	8.16 (6.08, 14.07)	0.950
CRP (mg/dl)	0.46 (0.20, 0.68)	0.46 (0.20, 0.69)	0.46 (0.19, 0.62)	0.920

For MRproANP and MRproADM, the percentages showing at least the optimal cut-off values determined by ROC analysis are additionally shown. *P*-values refer to the comparison of the two groups by the Mann–Whitney U-test or Fisher’s exact test. *MRproANP* midregional pro atrial natriuretic peptide, *MRproADM* midregional adrenomedullin, *NT-proBNP* B-type natriuretic peptide, *COPAVP* copeptin, *RAGE* soluble receptor for advanced glycation end products, *HS-Troponin* high-sensitivity troponin, *TNF* tumor necrosis factor alpha, *IL-6* Interleukin 6, *IL-8* Interleukin 8, *CRP* C-reactive pro

Numbers in bold represent *p* ≤ 0.05

### Association of biomarkers with vertebral fractures

In order to derive potentially useful cut-off values, ROC analyses were performed for MRproANP and MRproADM. These yielded 65 nmol/l for MRproANP (AUC: 0.673; 95%CI: 0.606, 0.740; *p* < 0.001) and 0.60 pg/mL for MRproADM (AUC: 0.611; 95%CI: 0.540, 0.682; *p* = 0.004) as optimal values. The optimal value for age was 67 years (AUC: 0.665; 95%CI: 0.599, 0.731; *p* < 0.001), corresponding to 70 years at the time of the CT scans.

**Fig. 3:**
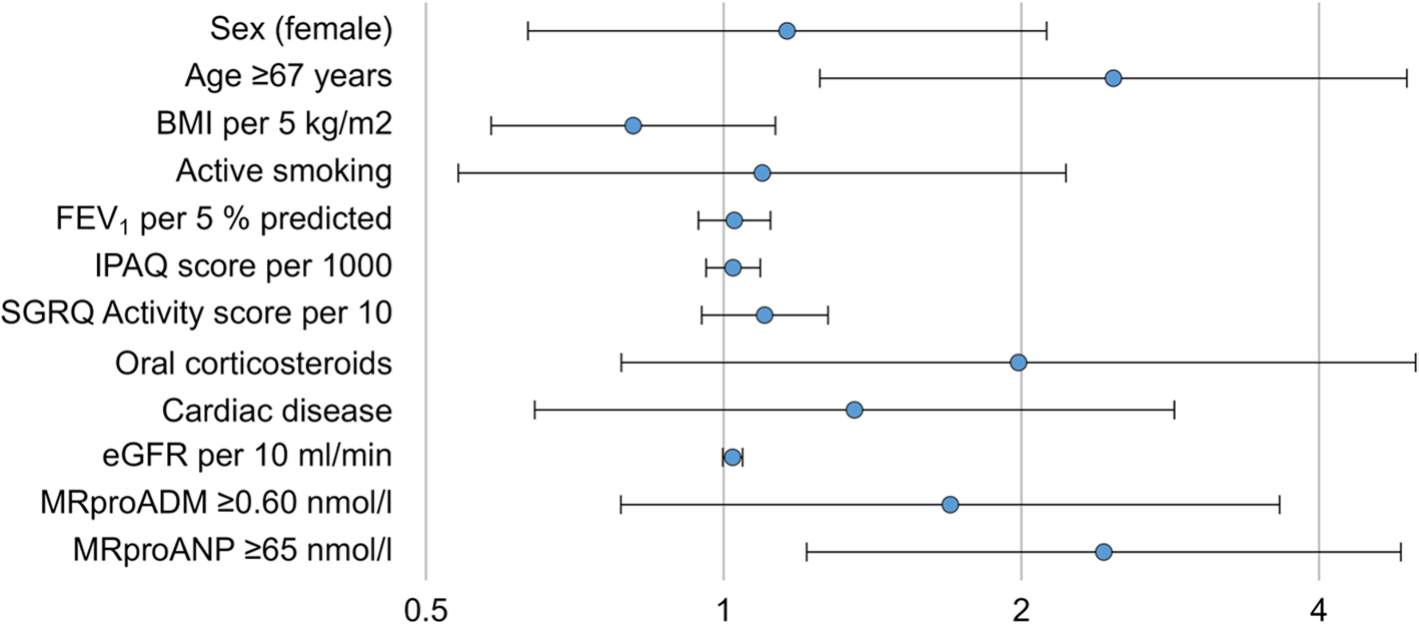
Results of the binary logistic regression analysis with the presence of vertebral fractures as outcome variable. The plot shows the odds ratios with their corresponding 95% confidence intervals on a logarithmic scale corresponding to the numerical values given in Table 3. BMI = body mass index, FEV_1_ = forced expiratory volume in 1 s, IPAQ = International Physical Activity Questionnaire, SGRQ = St George’s Respiratory Questionnaire, Cardiac disease = either coronary artery disease or heart failure, eGFR = estimated glomerular filtration rate, MRproANP = midregional pro-atrial natriuretic peptide, MRproADM = midregional pro-adrenomedullin. Please note that all predictors refer to Visit V1, as the values of MRproANP and MRproADM were assessed in blood samples obtained at this visit, while the outcome of fractures refers to Visit V4 about 3 years after Visit V1. Please note that some of the predictors were re-scaled in order to obtain odds ratios that were easier to interpret and distinguish from the reference value

For logistic regression analysis, we took age, sex, BMI, active smoking, the degree of airway obstruction (FEV_1_% predicted), self-reported physical activity (IPAQ), reported limitations of activity (SGRQ Activity), the diagnosis of either coronary artery disease or heart failure or both, and renal function in terms of eGFR as potentially relevant confounders. When using these variables as well as MRproADM and MRproANP as predictors, a value of ≥ 65 nmol/l for MRproANP was significantly (*p* = 0.012) associated with the presence of vertebral fractures, besides age (*p* = 0.009); see Fig. 3 and the numerical results shown in Table 3.

**Table 3:** Results of the binary logistic regression analysis with the presence of vertebral fractures as outcome variable

Variable	Regression coefficient B	SE of regression coefficient	Odds ratio Exp (B)	95%CI of Odds ratio		*P*-value
				**Lower**	**Upper**	
Sex (female)	0.148	0.308	1.159	0.634	2.121	0.632
Age ≥ 67 years	0.907	0.349	2.478	1.251	4.908	**0.009**
BMI per 5 kg/m^2^	-0.210	0.169	0.810	0.582	1.128	0.212
Active smoking	0.090	0.361	1.094	0.539	2.219	0.804
FEV_1_ per 5% predicted	0.025	0.043	1.025	0.943	1.115	0.556
IPAQ score per 1000	0.022	0.032	1.022	0.960	1.089	0.489
SGRQ Activity score per 10	0.010	0.008	1.010	0.995	1.025	0.203
Oral corticosteroids	0.687	0.472	1.987	0.788	5.009	0.145
Cardiac disease	0.305	0.380	1.356	0.644	2.856	0.422
eGFR per 10 ml/min	0.209	0.116	1.233	0.982	1.547	0.071
MRproADM ≥ 0.60 nmol/l	0.528	0.391	1.695	0.787	3.649	0.177
MRproANP ≥ 65 nmol/l	0.885	0.353	2.423	1.213	4.840	**0.012**

*95% CI* 95% confidence interval, *SE* standard error, *BMI* body mass index, *FEV*_*1*_ forced expiratory volume in 1 s, *IPAQ* International Physical Activity questionnaire, *SGRQ* St George’s Respiratory Questionnaire, Cardiac disease = either coronary artery disease or heart failure, *eGFR* estimated glomerular filtration rate, *MRproANP* midregional pro atrial natriuretic peptide, *MRproADM* midregional adrenomedullin. Please note that all predictors refer to Visit V1, as the values of MRproANP and MRproADM were assessed in blood samples obtained at this visit, while the outcome of fractures refers to Visit V4 about 3 years after Visit V1. Some of the predictors were re-scaled in order to obtain odds ratios that were easier to interpret

Numbers in bold represent *p* ≤ 0.05

### Association of biomarkers with vertebral density

To determine the association with TH12 vertebral mean attenuation as marker of BMD-CT, the same set of predictors was used as for fractures, and the results are shown in Table 4. Current smoking (*p* = 0.025) was associated with lower BMD-CT, and there were trends for age and SGRQ activity score, but these were not significant. While MRproADM was not statistically significant, a value of ≥ 65 nmol/l of MRproANP was associated with a reduction (*p* < 0.001) by about 19 HU, corresponding to a decrease of density by about 14% compared to the median value of the study population (Table 1).

**Table 4:** Results of the linear regression analysis with the TH12 vertebral mean attenuation as outcome variable

Variable	Regression coefficient B	SE of regression coefficient	95%CI of coefficient B		*P*-value
			**Lower**	**Upper**	
Sex (female)	2.318	4.684	-6.894	11.531	0.621
Age ≥ 67 years	-10.310	5.348	-20.828	0.209	0.055
BMI per 5 kg/m^2^	0.065	2.522	-4.895	5.025	0.979
Active smoking	12.040	5.336	1.545	22.534	**0.025**
FEV_1_ per 5% predicted	-0.324	0.639	-1.580	0.932	0.612
IPAQ score per 1000	-0.239	0.490	-1.202	0.724	0.626
SGRQ Activity score per 10	-0.209	0.111	-0.428	0.009	0.061
Oral corticosteroids	-0.298	8.374	-16.768	16.172	0.972
Heart failure	0.060	6.488	-12.701	12.821	0.993
eGFR per 10 ml/min	-1.379	1.752	-4.826	2.068	0.432
MRproADM ≥ 0.60 nmol/l	-5.440	5.443	-16.145	5.266	0.318
MRproANP ≥ 65 nmol/l	-18.526	5.325	-29.000	-8.053	** < 0.001**

*95%CI* 95% confidence interval, *SE* standard error, *BMI* body mass index, *FEV*_*1*_ forced expiratory volume in 1 s, *IPAQ* International Physical Activity questionnaire, *SGRQ* St George’s Respiratory Questionnaire, Cardiac disease = either coronary artery disease or heart failure, *eGFR* estimated glomerular filtration rate, *MRproANP* midregional pro atrial natriuretic peptide, *MRproADM* midregional adrenomedullin. Please note that all predictors refer to Visit V1, as the values of MRproANP and MRproADM were assessed in blood samples obtained at this visit, while the outcome of fractures refers to Visit V4 about 3 years after Visit V1. Some of the predictors were re-scaled in order to obtain regression coefficients that were easier to interpret

Numbers in bold represent *p* ≤ 0.05

## Discussion

The present study investigated vertebral fractures and bone mineral density derived from chest CT scans in patients with COPD. Its aim was to reveal whether these outcomes are associated with MRproANP and MRproADM that can influence overall fluid load and ion balance and thus potentially bone integrity, beyond their role as markers of cardiovascular risk. Indeed, the presence of vertebral fractures was associated with elevated levels of MRproANP. This association was maintained when taking into account covariates such as activity limitations and the presence of cardiac comorbidities. In univariate analyses, there were also associations with MRproADM but these disappeared after adjustment. When choosing TH12 mean attenuation in the CT as a marker of bone mineral density, results were consistent with those obtained for fractures, and were again related to MRproANP levels. Taken together, our findings suggest that MRproANP that we previously identified as a predictor of mortality risk in recently diagnosed patients with mild to moderate COPD [13], has the additional property being a marker of bone alterations related to osteoporosis. This observation could be beneficial for clinical practice in order to use biomarkers as efficiently as possible.

Our analysis was motivated by the findings of Johansson et al. [34], who longitudinally assessed the association between cardiac markers and the incidence of vertebral, pelvic and limb fractures in a large population-based prospective cohort and found that elevated levels of MR-proADM and MR-proANP predicted fractures in older adults. The proposed mechanism was that syncopal events and falls caused by hemodynamic and autonomic effects linked to these hormones played a role and that markers of endothelial dysfunction could also indicate microvascular dysfunction in bone tissue, leading to bone fragility [34].

We directly assessed bone structure in terms of vertebral integrity in chest CT scans, focusing on patients with COPD. This focus was based on the hypothesis that in COPD an association between bone integrity and cardiovascular markers might be particularly likely, due to systemic inflammation and the high prevalence of cardiovascular comorbidities. This was confirmed in univariate analyses for MRpro ANP and MRpro ADM, while in adjusted analyses only MRproANP remained as robust and significant marker. Considering, that serum MRproANP concentrations are < 50 pmol/l in healthy individuals [37, 38], the optimal cut-off value of 65 nmol/l was only slightly elevated. Despite this, the corresponding odds ratio for fractures was 2.3 in our opinion high enough to be clinically considered.

MRproANP is generally considered as a marker of heart failure [39] and belongs to the family of natriuretic peptides (NPs), which are structurally related, but functionally different hormones with multiple functions, including the regulation of blood pressure, water–mineral balance, and various metabolic processes [40]. There is also evidence that NPs play a role in bone metabolism [41, 42], based on the observations that B-type natriuretic peptide was associated with lower BMD and incident osteoporosis in peritoneal dialysis patients [43], kidney transplant recipients [44] and type 2 diabetic patients [45]. The differences between the various NPs were also highlighted in our data by the finding that NT-proBNP was not associated with vertebral fractures or decreased BMD.

In consistence with clinical observations, our study found vertebral fractures to be fairly common in patients with COPD. Chest CT showed fractures of the thoracic spine in 18% of men and 15.9% of women, with no major differences related to sex, although osteoporosis is usually more often diagnosed in women. Moreover, not many patients had a previous diagnosis of osteoporosis in our study. The findings suggest that osteoporosis is underdiagnosed in COPD and that its diagnosis might depend on gender-specific attention. The results are consistent with findings from the COPDGene cohort study, which showed a slightly but significantly higher risk of low vertebral BMD and more fractures in male compared to female smokers. In our study, case numbers were too low to allow for a similar analysis, and in addition our focus was on the potential role of the cardiovascular markers. It might, however, be of great interest to re-analyze the previous data, if possible, in order to validate our findings.

There was no association between fractures and quality of life, COPD symptoms or physical activity, probably due to the fact that the vertebral alterations were on the level of being non-symptomatic and the lung disease exerted an overwhelming effect on these outcomes. While vertebral fractures were associated with higher age and a higher level MRproANP, vertebral bone density showed further associations, such as the Activity component of the SGRQ being correlated with reduced BMD-CT. In contrast, active smoking was associated with higher density, probably due to the fact that in COSYCONET active smokers with COPD are of lower age and less clinical severity compared to ex-smokers [46]. The finding that a reduced eGFR was also associated with higher vertebral density is more difficult to explain and may reflect a selection effect. Moreover, chronic kidney disease may be linked to low density in the hip but not the spine [47].

It is commonly assumed that patients with COPD experience an increased risk of osteoporosis and osteoporotic fractures of the spine compared to the general population [2]. For this there are established risk scores such as the FRAX online tool, which, however, does not appear to be predictive in patients with COPD [48]. In our study, vertebral bone density (BMD-CT) showed a significant difference between the groups with and without vertebral fractures but with a large overlap. Thus, the assessment of BMD-CT alone was not sufficient to reliably assess the presence of fractures, which might be preferable as indicator of osteoporosis. Both measures, however, had in common to be associated with MRproANP.

### Limitations

The cross-sectional analysis allows for statements on correlations, but per se no causal inferences. CT assessments were performed about 3 years after the assessments of the variables used as statistical predictors. This choice was due to the fact that cardiovascular biomarker values were available only upon inclusion into COSYCONET. When tentatively using values assessed at Visit 4 for all predictors except the cardiovascular markers, the results for MRproANP were maintained. Moreover, the assessment of comorbidities was based on patients’ reports of physician-made diagnoses, not on systematic testing using standardized methods. This probably led to underdiagnosis of osteoporosis, which was, however, without consequences, as we excluded this diagnosis from the panel of predictors to avoid trivial results. We also could not determine incidence rates. Moreover, only vertebral fractures of the thoracic spine were assessed since this was the only part covered with routine chest CT. The available literature suggests that osteoporosis-defining fractures of the ribs and outside the region covered by chest CT, e.g. in the cervical or lumbar spine, pelvis or hip, may also be associated with elevated levels of cardiovascular blood markers [34]. This aspect may warrant attention in future studies on this topic.

## Conclusion

In patients with COPD, the presence of vertebral fractures of the thoracic spine and a reduced vertebral density on chest CT were statistically associated with known risk factors such as age but additionally with elevated blood levels of midregional pro-atrial natriuretic peptide (MRproANP), which is commonly considered as cardiovascular marker. This may be explained by the pleiotropic role of MRproANP and is consistent with previous findings in the general population reporting its association with fractures in the elderly. Taken together with its cardiovascular role, the observations suggest a role for MRproANP in COPD as part of a comprehensive panel of blood biomarkers for the assessment of outcome- limiting multimorbidity even at subclinical stages. Further research on the mechanisms by which MRproANP could affect bone mineralization seems worthwhile.

## Data Availability

No datasets were generated or analysed during the current study.

## Abbreviations

6-MWD: 6-minute walk distance
95%CI: 95% confidence interval
AIRC: AI-Rad Companion Chest CT
AUC: Area under the curve
BMD: Bone mineral density
BMD-CT: Bone density on CT
BMI: Body mass index
CAT: COPD assessment Test
COPD: Chronic obstructive pulmonary disease
COSYCONET: COPD and Systemic Consequences - Comorbidities Network
CRP: C-reactive protein
DLCO: Diffusing capacity of the lung for carbon monoxide
DXA: Dual-energy X-ray absorptiometry
eGFR: Estimated glomerular filtration rate
FEV_1_: Forced expiratory volume in 1 second
FVC: Forced vital capacity
GLI: Global Lung Function Initiative
GOLD: Global Initiative for Chronic Obstructive Lung Disease
HS-troponin: High-sensitivity troponin
HU: Hounsfield-Units
IL-6: Interleukin 6
IL-8: Interleukin 8
IPAQ: International Physical Activity Questionnaire
mMRC: Medical Research Council
MRproADM: Midregional pro-adrenomedullin
MRproANP: Mid-regional pro-atrial natriuretic peptide
NP: Natriuretic peptide
NT-proBNP: N-terminal pro-B-type natriuretic peptide
RAGE: Soluble receptor for advanced glycation end products
SE: Standard error
SGRQ: St Georges’s Respiratory Questionnaire
TH12: 12^th^ segment in the thoracic part of the spine (*pars thoracica medullae spinalis*)
TNF: Tumor necrosis factor alpha
